# *Bartonella quintana* Infections in Captive Monkeys, China

**DOI:** 10.3201/eid1709.110133

**Published:** 2011-09

**Authors:** Ruting Huang, Qiyong Liu, Genping Li, Dongmei Li, Xiuping Song, Richard J. Birtles, Fan Zhao

**Affiliations:** Author affiliations: Chinese Center for Disease Control and Prevention, Beijing, People’s Republic of China (R. Huang, Q. Liu, D. Li, X. Song, F. Zhao);; Fengtai District Center for Disease Control and Prevention, Beijing (R. Huang);; Beijing Administration Office of Laboratory Animal, Beijing (G. Li);; University of Salford, Greater Manchester, UK (R.J. Birtles)

**Keywords:** Bartonella quintana, monkeys, epidemiology, experimental animals, bacteria, zoonoses, China, dispatch

## Abstract

*Bartonella quintana* has been considered to be specifically adapted to humans. Our isolation of the organism from 2 of 36 captive rhesus macaques in China and finding antibodies against *B. quintana* in 12 of 33 indicates that the reservoir hosts of *B. quintana* may include primates other than humans.

*Bartonella* spp. are bacterial hemoparasites with a wide variety of mammalian hosts as reservoirs. Several members of the genus are pathogens of medical and veterinary significance. Most human infections are zoonoses; however, 2 *Bartonella* spp., *B. bacilliformis* and *B. quintana*, are considered to be specifically adapted to humans (*1*). Although, like other members of the genus, both species generally cause chronic intraerythrocytic bacteremia of little clinical consequence to their reservoir hosts, both are also associated with illness and death (*1*).

*B. quintana* infections most frequently cause recurrent fever and pretibial pain (trench fever), endocarditis, and bacillary angiomatosis (*2*). *B. quintana* is transmitted by the human body louse (*Pediculus humanis humanis*), which thrives in the absence of basic sanitation and hygiene; hence, infections are most frequently associated with persons who are homeless or affected by social or civil unrest (*3*), although they are increasingly encountered in rural communities in developing regions of the world (*4**,**5*).

Some reports suggest that humans are not the only reservoir hosts of *B. quintana.* In 2005, *B. quintana* was recovered from the blood of a cynomolgus monkey (*Macaca fascicularis*) imported into the United States from Southeast Asia (*6*). In addition, infections resulting in chronic bacteremia have been experimentally established in rhesus macaque monkeys (*Macaca mulatta*) inoculated with *B. quintana* isolates from infected humans (*7**,**8*).

## The Study

In 2009, blood samples were collected from 36 apparently healthy rhesus macaque monkeys housed in a biologic research institute in Beijing, People’s Republic of China. Breeding, delivery, and raising the young monkeys took place in an outdoor arena that housed a large group of animals; the monkeys were later moved indoors into isolation. Samples were stored at −80°C, thawed, and then plated (400 µL of each) onto 5% (vol/vol) sheep blood–enriched Columbia agar and incubated at 37°C in 5% CO_2_ for as long as 45 days. After 15 days of incubation, 2 plates yielded putative *Bartonella* spp. colonies. One plate, inoculated with blood from a 3-year-old female monkey (S13), yielded 13 colonies; the other, inoculated with blood from a 2-year-old male monkey (M22), yielded 2 colonies. Microscopic examination of Gram-stained smears of these colonies revealed small, pleomorphic, gram-negative bacteria; their identity was confirmed by using molecular methods.

DNA extracts were prepared from colonies by using a QIAamp tissue kit (QIAGEN, Hilden, Germany) and incorporated into *Bartonella* genus–specific PCRs selective for fragments of the 16S rRNA–encoding gene, the 16S–23S rDNA intergenic spacer region (ISR), *gltA, ribC*, and *ftsZ* (*9**–**12*). Extraction controls (water only) were concurrently prepared with DNA extracts and incorporated into each PCR along with a reagent-only (no DNA) negative control and a reaction-positive control (*B. henselae* DNA). In all assays, DNA extracts and positive controls yielded amplification products, whereas extraction and reagent-only controls did not.

The nucleotide base sequences of all amplification products were determined. Analysis of sequence data indicated that the 2 isolates possessed indistinguishable *gltA*, 16S rDNA, and ISR sequences but that their *ribC* and *ftsZ* sequences differed from each other by 1 bp mutation. Comparison of these data with those available for validated *Bartonella* spp. indicated that the sequences of isolates S13 and M22 were most similar to those of *B. quintana* (*ribC* 97%, *gltA* 98%, ISR 98%, *ftsZ* 99%, and 16S rDNA 100% similarity). These similarity values are higher than those previously proposed as thresholds for delineation of *Bartonella* spp (*13*), suggesting that the isolates S13 and M22 were strains of *B. quintana*.

We inferred the phylogenetic positions of the 2 isolates within the genus from alignment of concatenated sequence data (all 5 loci). In this inference, isolates S13 and M22 grouped together and formed a well-supported tight cluster with *B. quintana* and no other *Bartonella* spp. (Figure). The phylogenetic distances between *B. quintana*, isolate S13, and isolate M22 were much shorter than those between, for example, *Bartonella vinsonii* subsp., supporting the classification of isolates S13 and M22 as strains of *B. quintana*.

**Figure:**
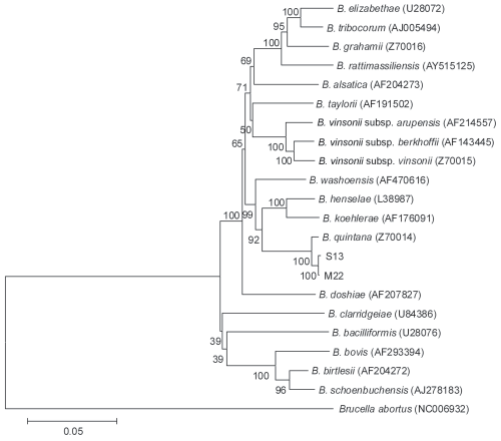
Phylogenetic dendrogram of *Bartonella* spp., inferred from alignment of concatenated sequence data (4,007 bp) by using a maximum-likelihood algorithm, within the MEGA4 suite (www.megasoftware.net). The strength of proposed branching orders was tested by bootstrapping (1,000 replicates), and the percentage of samples supporting the proposed branching order are indicated at each node. Sequence data from the 5 loci studied for isolates S13 (from captive 3-year-old female rhesus macaque monkey, China) and M22 (from captive 2-year-old male rhesus macaque monkey, China) were submitted to GenBank under accession nos. HQ014621 (S13 16S rDNA), HQ014622 (S13 ISR), HQ014623 (S13 *ftsZ*), HQ014624 (S13 *gltA*), HQ014625 (S13 *ribC*), HQ014628 (M22 16S rDNA), HQ014629 (M22 ISR), HQ014630 (M22 *ftsZ*), HQ014627 (M22 *gltA*), and HQ014626 (M22 *ribC*). Scale bar indicates nucleotide substitutions per site.

Subsequently, by using a commercial indirect immunofluorescence antibody test kit (Euroimmun, Lubeck, Germany), we found antibodies against *B. quintana* in 33 monkey serum samples. Serum from 12 monkeys yielded a high titer (>320). Serum was available from only 1 of the culture-positive animals, M22, and this sample had a positive titer of 320. Overall, we found *B. quintana* seroprevalence to be significantly lower in sexually mature monkeys (χ^2^ = 6.034, p = 0.014), but we found no significant correlation between seroprevalence and age or gender. We obtained the *B. quintana* isolates ≈6 weeks after blood collection and immediately attempted to resample the 2 infected monkeys. We were able to obtain blood from monkey M22 only; this sample did not yield further isolates. However, the animal remained seropositive, with an anti–*B. quintana* titer of 320.

## Conclusions

What is particularly intriguing about the recovery of *B. quintana* from the blood of 2 apparently healthy rhesus macaques is that the monkeys were not members of a natural wild population; rather, they had been bred in captivity in suburban Beijing and held in enclosures apart from other animals. This recovery suggests that *B. quintana* was being maintained in the colony or that monkeys acquired infection from the only other animals they had contact with: humans. High *B. quintana* seroprevalence in the colony, higher for immature than older animals, suggests that other monkeys had also been exposed to the bacterium, although the true meaning of these findings is unclear, particularly because the specificity of the immunoassay in monkeys in unknown.

Examination of the monkeys from which blood was collected failed to reveal any ectoparasites, and officials at the animal facility reported never seeing ectoparasites on the monkeys. Thus, we are no closer to identifying which species of arthropod, if any, serves as a vector for the monkey-associated *B. quintana* infection.

Although isolates S13 and M22 should best be considered strains of *B. quintana*, they are, nonetheless, apparent outliers within the currently recognized diversity of the species. Previous work surveying genetic diversity among 16 human-associated *B. quintana* isolates of diverse provenance (*14*) encountered no *gltA* sequence dissimilarity and only 0.2% *ftsZ* sequence dissimilarity, whereas isolates S13 and M22 displayed >1% dissimilarity with the *B. quintana* type strain at both loci. This level of sequence dissimilarity is similar to that reported between the *B. quintana* type strain and the isolate from a cynomolgus monkey (*6*). Unfortunately, because those data were not deposited in GenBank, we were unable to directly compare our sequence data with those previously reported for this *B. quintana* strain. Such comparisons might indicate the existence of nonhuman primate–adapted genotypes within the species.
